# Anatomical Atlas—Illustrative of the Structure of the Human Body

**Published:** 1844-04

**Authors:** 


*Anatomical Atlas—Illustrative of the structure of the human body.* By HENRY H. SMITH, M. D., Fellow of the College of Physicians, Member of the Philadelphia Medical Society, &c. Under the supervision of WILLIAM E. HORNER, M. D., Professor of Anatomy in the University of Pennsylvania, &c. Philadelphia: Lee & Blanchard, 1844.

This work, of which the first number, embracing the bones and ligaments, is now before us, is published for the purpose of "forming a set of Plates as an accompaniment to the text work" of Dr. Horner, of which a new edition with many additions has lately been issued. Most of the figures are selections from different works, but a part are from original drawings of preparations in the Museum of the University. There are several representing the tissues of bone and cartilage, viewed with and without the microscope, while the form of all the bones and the situations and attachments of the ligaments are perfectly exhibited. The engravings are excellent, the form and size of the work are such as to render its use very convenient. The Atlas is to be completed in five Nos., price $1,00 each, and will form a useful accompaniment, not only to Dr. Horner's, but to any other full and recent system of Anatomy. D. B.

